# Supplementary material for: Assembly and phosphoregulatory mechanisms of the budding yeast outer kinetochore KMN complex
Source: J Cell Biol. 2026 Apr 9;225(5):e202506015. doi: 10.1083/jcb.202506015 (PMC13065467; doi:10.1083/jcb.202506015)
Supplement: Table S1 — shows cryo-EM data collection, refinement, and validation statistics. [file jcb_202506015_tables1.docx]

| **Data collection and processing** | | | | | | |
| --- | --- | --- | --- | --- | --- | --- |
| Microscope | FEI Titan Krios 1 | | | | | |
| Voltage (keV) | 300 | | | | | |
| Magnification | 105,000 | | | | | |
| Detector | Gatan K3 | | | | | |
| Electron dose (e^-^/Å^2^) | 40 | | | | | |
| Pixel size (Å/pixel) | 0.825 | | | | | |
| Exposure rate (e^-^/pixel/s) | 15.6 | | | | | |
| Frames/micrograph (N) | 40 | | | | | |
| Automation software | EPU | | | | | |
| Micrographs collected (N) | 26,390 | | | | | |
| Micrographs used (N) | 26,390 | | | | | |
| Defocus (μm) | -1.4 to -3.0 | | | | | |
| Magnification | 105,000 | | | | | |
| Total extracted particles (N) | 2,592,458 | | | | | |
| **Cryo-EM reconstructions** | | | | | | |
|  | KMN junction consensus map | KMN junction composite map | KMN junction apex body 1 | KMN junction base body 1 | KMN junction consensus map (head 2) | KMN junction base body 2 |
| Figure | S2Dii | S2Dii | S2E | S2F | S4A | S4C |
| Accession codes | EMD-54576  PDB N/A | EMD-54579  PDB 9S4Q | EMD-54578  PDB N/A | EMD-54577  PDB N/A | EMD-54602  PDB 9S5N | EMD-54586  PDB 9S53 |
| Final particles (N) | 113,688 | 113,688 | 113,688 | 113,688 | 18,160 | 18,160 |
| Point group | C1 | C1 | C1 | C1 | C1 | C1 |
| Resolution global (Å) | 6.0 | 4.8-4.9 | 4.8 | 4.9 | 7.2 | 6.5 |
| Resolution range local (Å) | ND | ND | 4.2-5.4 | 4.7-5.3 | ND | 5.7-7.3 |
| FSC threshold | 0.143 | 0.143 | 0.143 | 0.143 | 0.143 | 0.143 |
| *Map sharpening method* | RELION | | | | | |
| **Refinement** | | | | | | |
| *Software* | Phenix | | | | | |
| Resolution limit (Å) | N/A | 4.9 | N/A | N/A | 7.2 | 6.5 |
| Protein atoms (N) |  | 19,480 |  |  | 21,005 | 8,388 |
| RMSD bond length (Å) |  | 0.019 |  |  | 0.018 | 0.027 |
| RMSD bond angle (^o^) |  | 0.567 |  |  | 0.446 | 0.596 |
| *Model to map fit* | | | | | | |
| CC_mask | N/A | 0.6770 | N/A | N/A | 0.8221 | 0.8419 |
| CC_volume |  | 0.6899 |  |  | 0.8186 | 0.8423 |
| *Validation* | | | | | | |
| All-atom clash score | N/A | 9.81 | N/A |  | 15.77 | 34.52 |
| *Ramachandran plot* | | | | | | |
| Preferred (%) | N/A | 95.04 | N/A | N/A | 96.02 | 95.66 |
| Allowed (%) |  | 4.88 |  |  | 3.91 | 4.17 |
| Outliers (%) |  | 0.09 |  |  | 0.07 | 0.17 |

**Supplementary Table S1:** Cryo-EM data collection, refinement and validation statistics
