# Supplementary material for: Assembly and phosphoregulatory mechanisms of the budding yeast outer kinetochore KMN complex
Source: J Cell Biol. 2026 Apr 9;225(5):e202506015. doi: 10.1083/jcb.202506015 (PMC13065467; doi:10.1083/jcb.202506015)
Supplement: Table S2 — shows genotypes of S. cerevisiae strains used in this study. [file jcb_202506015_tables2.docx]

| Strain | Genotype |
| --- | --- |
| BY26972 (The Yeast Resource Genetic Resource Center, Japan) | *MAT* ***a*** *ade2-1 his3-11,15 trp1-1 leu2-3,112 can1-100 ura3-1∷ADH1 OsTIR1(Pmk200, URA3)* |
| Ynnt001 | *MAT* ***a*** *ade2-1 his3-11,15 trp1-1 leu2-3,112 can1-100 ura3-1∷ADH1 OsTIR1(Pmk200, URA3) MTW1-mAID_3_-FLAG_5_::G418* |
| Ynnt002 | *MAT* ***a*** *ade2-1 his3-11,15 trp1-1 leu2-3,112 can1-100 ura3-1∷ADH1 OsTIR1(Pmk200, URA3) NNF1-mAID_3_-FLAG_5_* |
| *MTW1* | *MAT* ***a*** *ade2-1 his3-11,15 trp1-1 leu2-3,112 can1-100 ura3-1∷ADH1 OsTIR1(Pmk200, URA3), MTW1-mAID_3_-FLAG_5­­_, HA_3_-MTW1::LEU2* |
| *Mtw1^ΔC^* | *MAT* ***a*** *ade2-1 his3-11,15 trp1-1 leu2-3,112 can1-100 ura3-1∷ADH1 OsTIR1(Pmk200, URA3), MTW1-mAID_3_-FLAG_5­­_, HA_3_-MTW1(Δ272-C)::LEU2* |
| Empty vector (LEU2) | *MAT* ***a*** *ade2-1 his3-11,15 trp1-1 leu2-3,112 can1-100 ura3-1∷ADH1 OsTIR1(Pmk200, URA3) MTW1-mAID_3_-FLAG_5_, Pleu2::LEU2* |
| *NNF1* | *MAT* ***a*** *ade2-1 his3-11,15 trp1-1 leu2-3,112 can1-100 ura3-1∷ADH1 OsTIR1(Pmk200, URA3), NNF1-mAID_3_-FLAG_5­­_, V5_3_-NNF1::TRP1* |
| *Nnf1^ΔC^* | *MAT* ***a*** *ade2-1 his3-11,15 trp1-1 leu2-3,112 can1-100 ura3-1∷ADH1 OsTIR1(Pmk200, URA3), NNF1-mAID_3_-FLAG_5­­_, V5_3_-NNF1(Δ180-C)::TRP1* |
| Empty vector (TRP1) | *MAT* ***a*** *ade2-1 his3-11,15 trp1-1 leu2-3,112 can1-100 ura3-1∷ADH1 OsTIR1(Pmk200, URA3), NNF1-mAID_3_-FLAG_5­­_, Ptrp1::TRP1* |

**Supplementary Table S2:** Genotypes of *S. cerevisiae* strains used within this study.
