# Supplementary figures and images for: Assembly and phosphoregulatory mechanisms of the budding yeast outer kinetochore KMN complex
Source: J Cell Biol. 2026 Apr 9;225(5):e202506015. doi: 10.1083/jcb.202506015 (PMC13065467; doi:10.1083/jcb.202506015)

Figure 2E

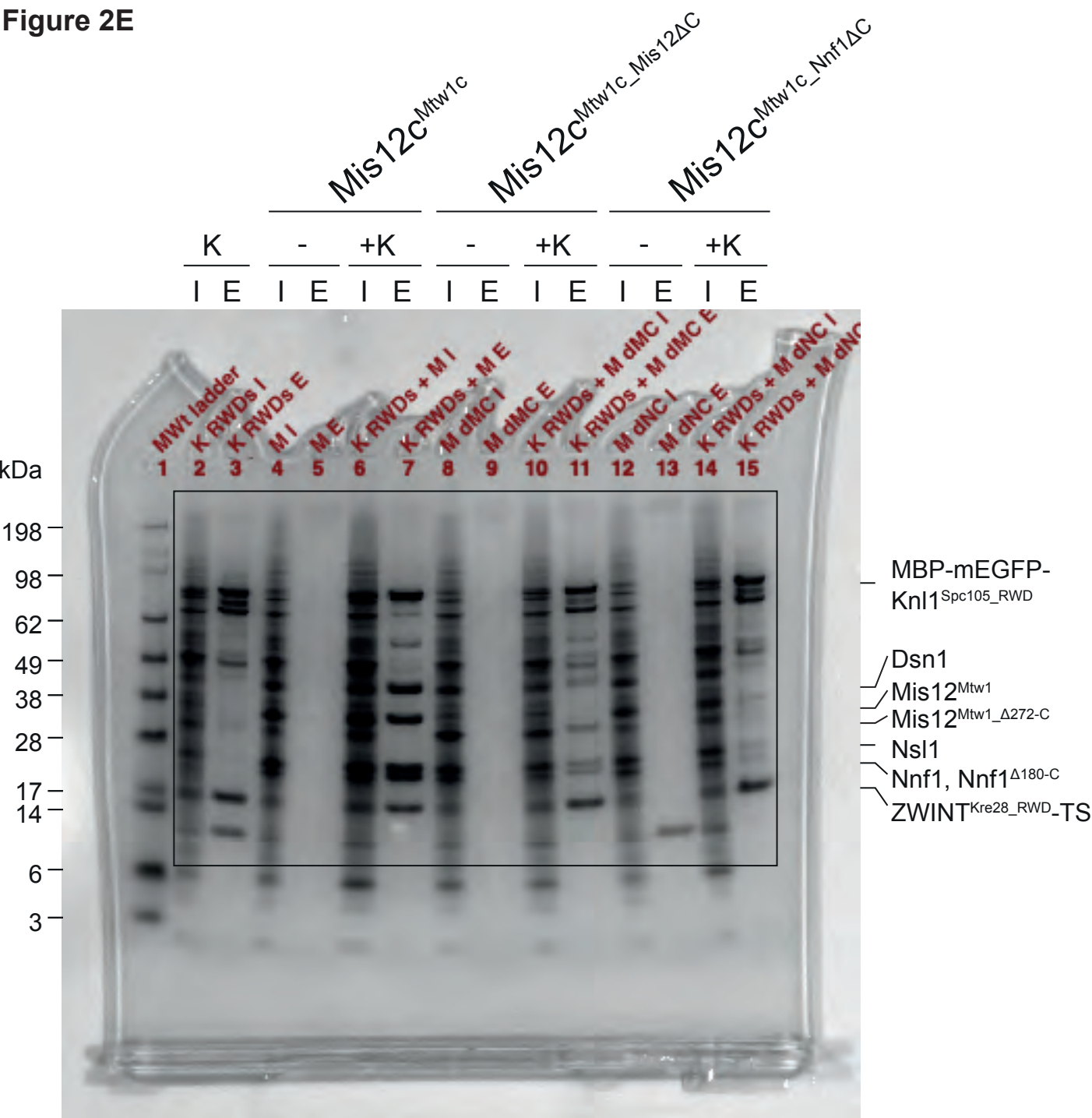

Supplement: SourceData F2 — is the source file for Fig. 2. [file jcb_202506015_sourcedataf2.pdf]

Figure 7Ci

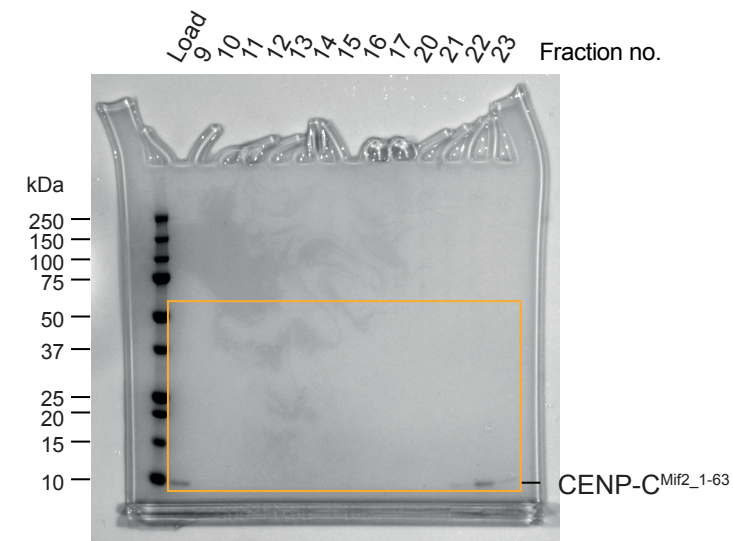

Figure 7Cii

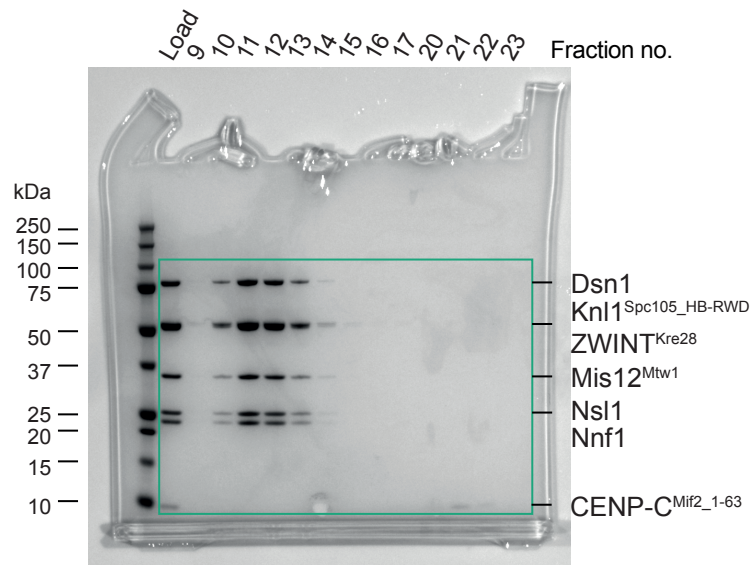

Figure 7Ciii

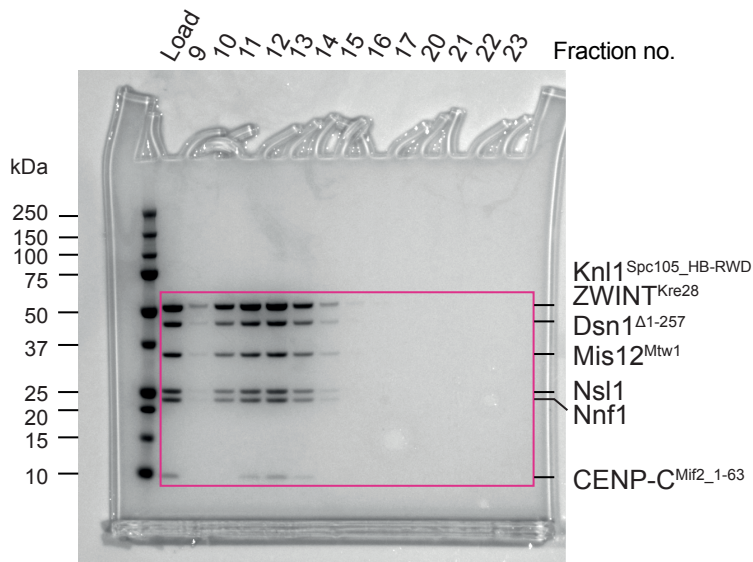

Figure 7Civ

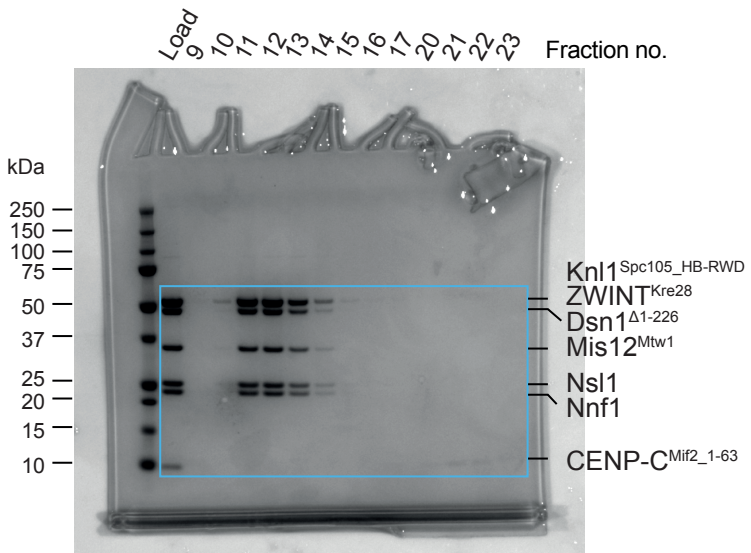

Figure 7Cv

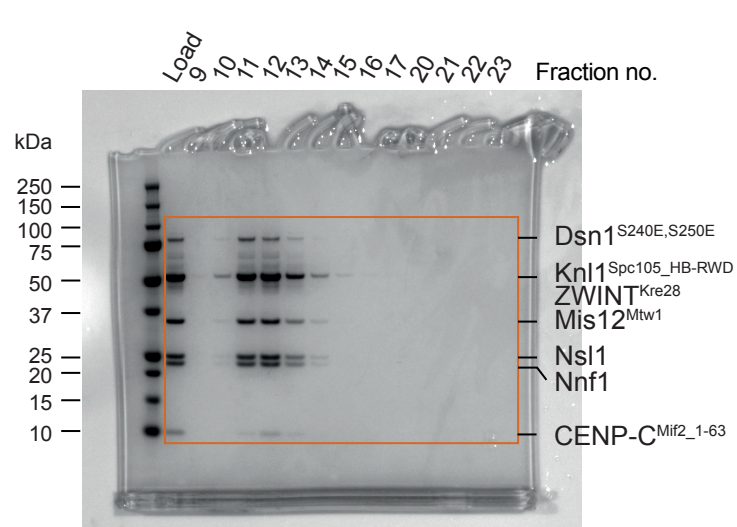

Figure 7Cvi

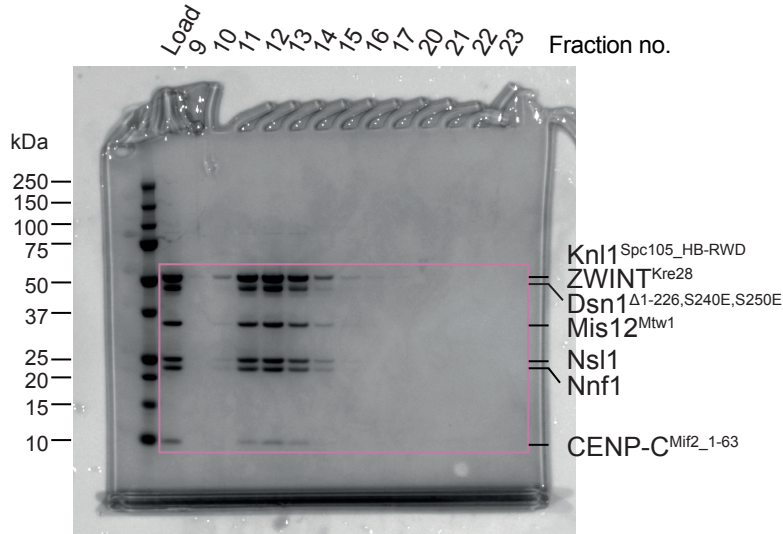

Supplement: SourceData F7 — is the source file for Fig. 7. [file jcb_202506015_sourcedataf7.pdf]

Figure 8F

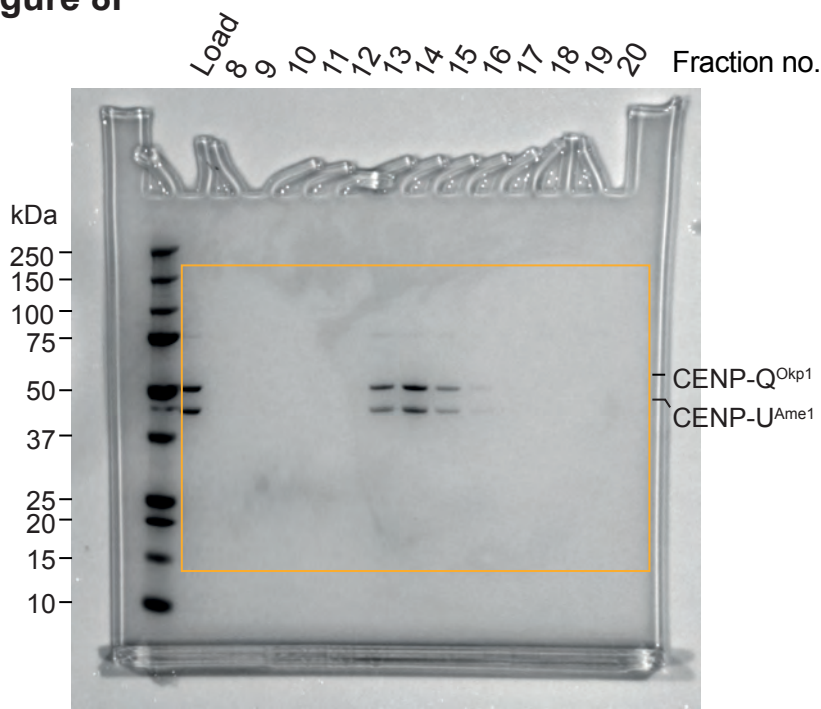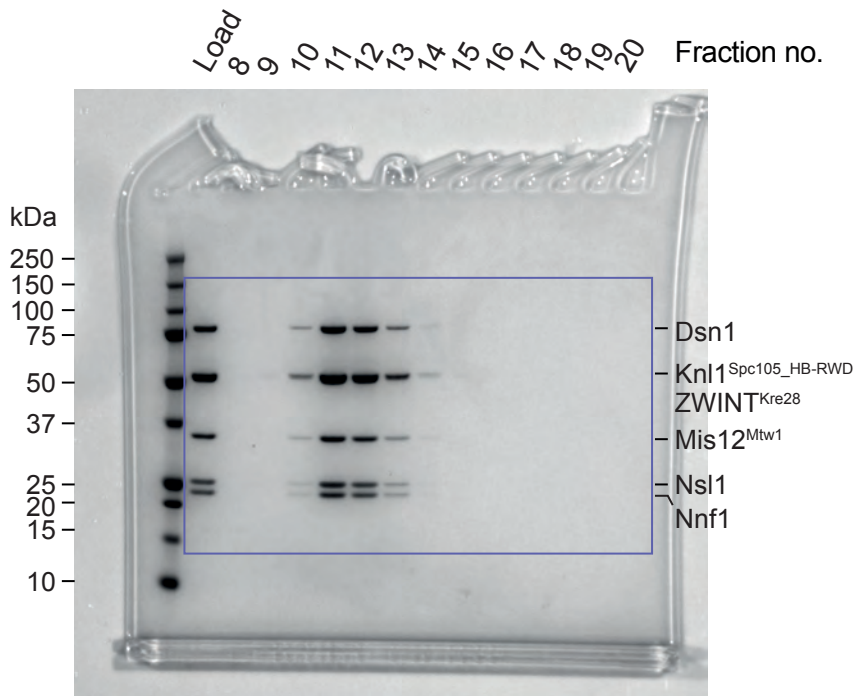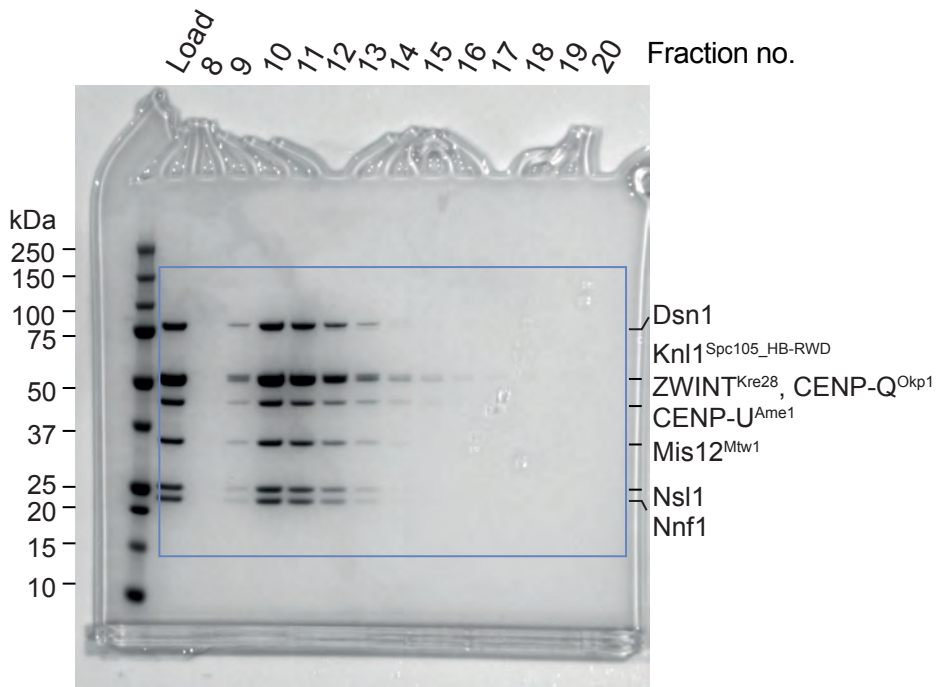

Supplement: SourceData F8 — is the source file for Fig. 8. [file jcb_202506015_sourcedataf8.pdf]

Figure S1C

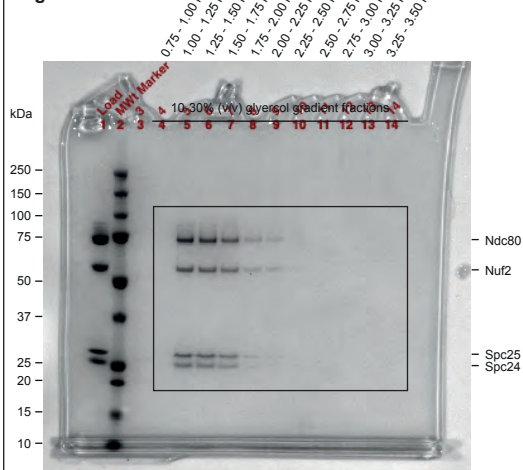

**Figure S1D**

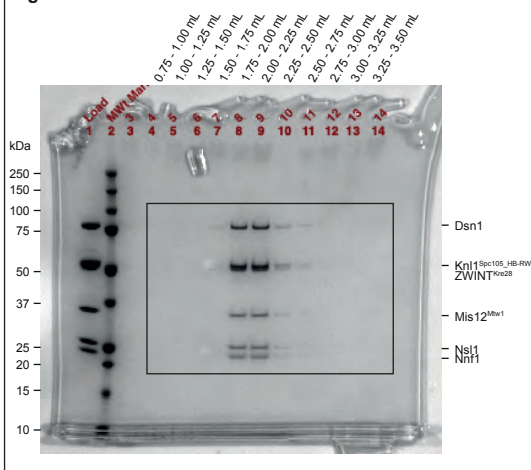

Figure S1E

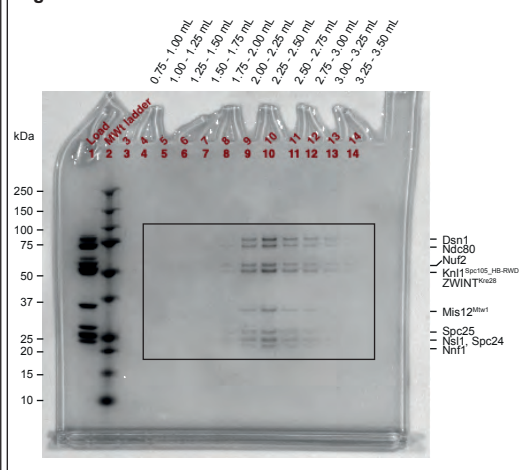

**Figure S1B**

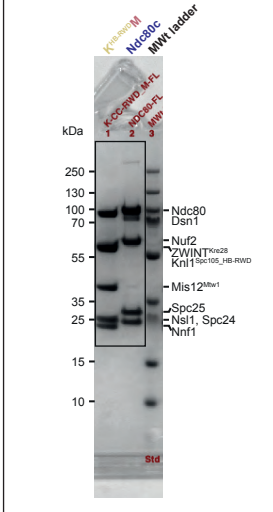

Figure S1F

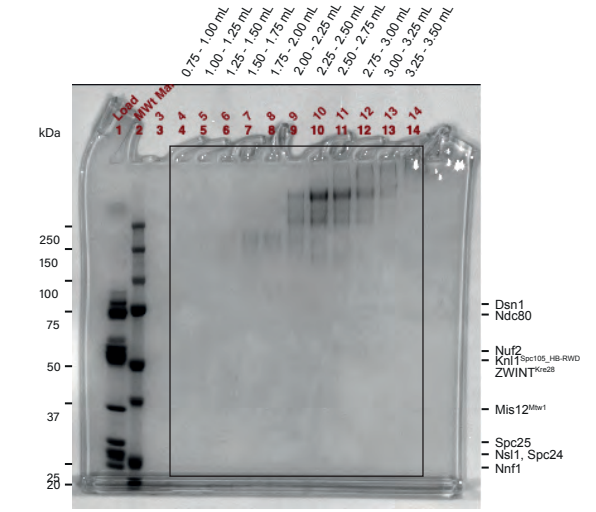

Figure S1G

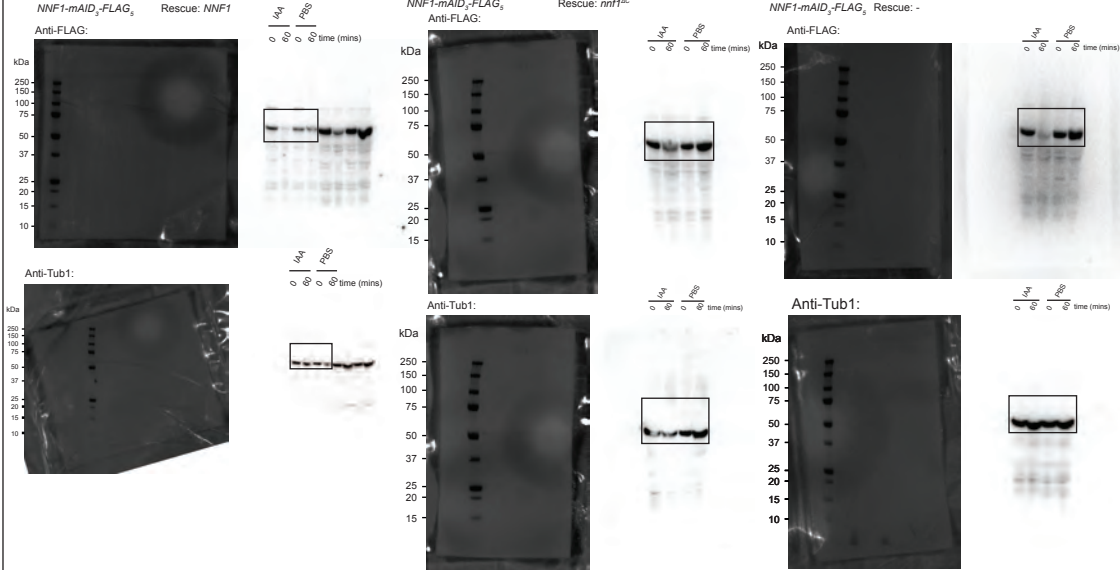

Figure S11

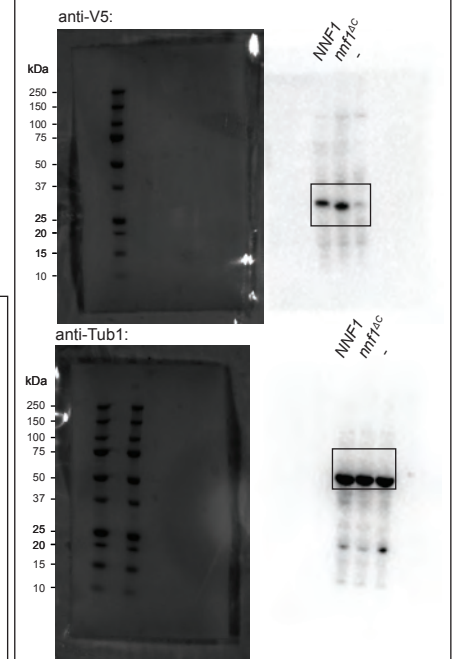

Figure S1J

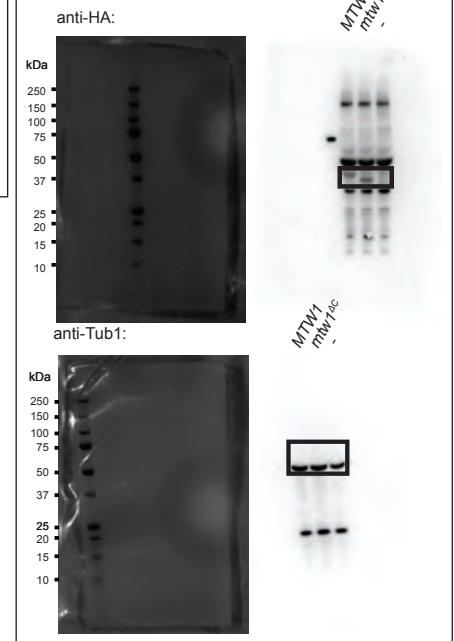

Figure S1H

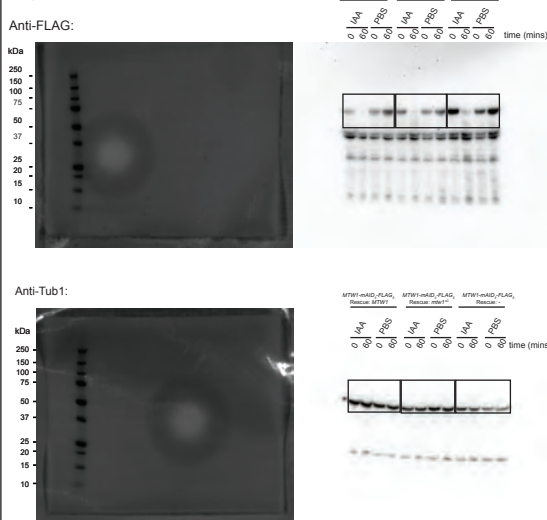

Supplement: SourceData FS1 — is the source file for Fig. S1. [file jcb_202506015_sourcedatafs1.pdf]
