# Supplementary material for: Assembly and phosphoregulatory mechanisms of the budding yeast outer kinetochore KMN complex
Source: J Cell Biol. 2026 Apr 9;225(5):e202506015. doi: 10.1083/jcb.202506015 (PMC13065467; doi:10.1083/jcb.202506015)

Figure S3B

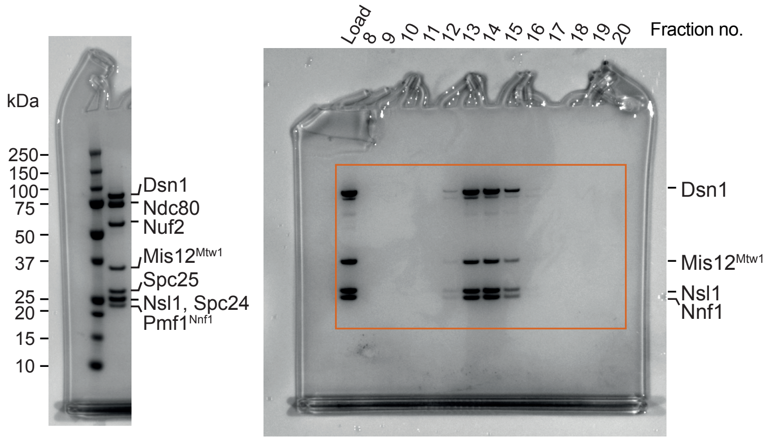

Neglected to load MWt marker, so molecular weight labels are from Figure S3D Ndc80c + Mis12c^{Mtw1c} which was run on the same type of gel (NuPage 4-12% Bis-Tris; ThermoFisher) in the same gel tank at the same time and has common bands.

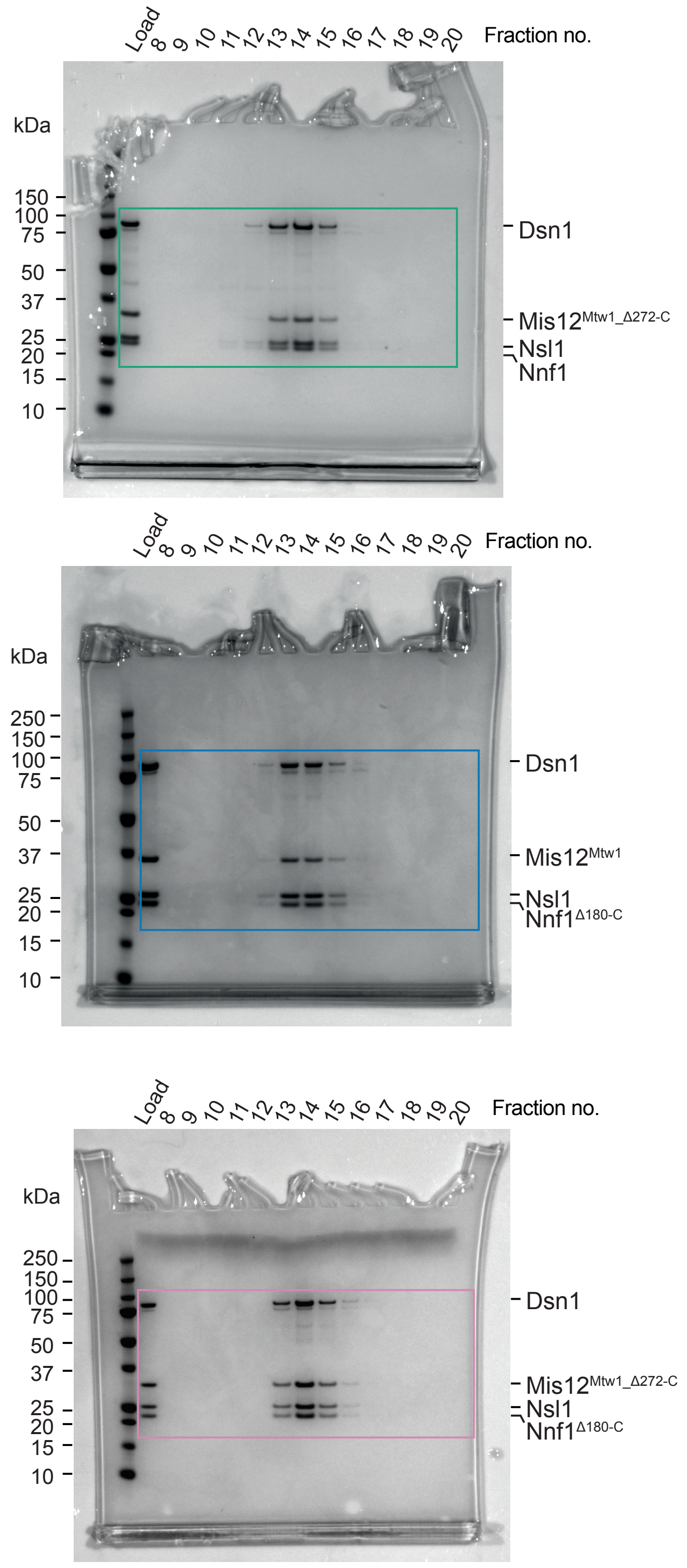

Figure S3D

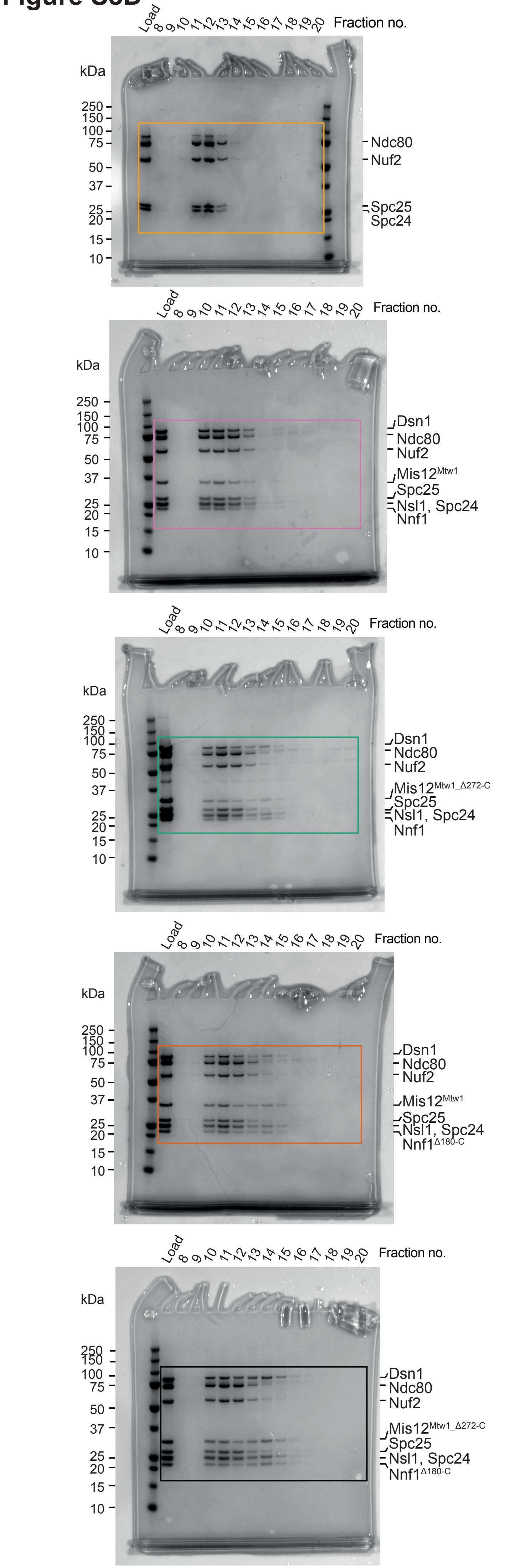

Supplement: SourceData FS3 — is the source file for Fig. S3. [file jcb_202506015_sourcedatafs3.pdf]
